# Adolescent health programming in India: a rapid review

**DOI:** 10.1186/s12978-020-00929-4

**Published:** 2020-06-03

**Authors:** Alka Barua, Katherine Watson, Marina Plesons, Venkatraman Chandra-Mouli, Kiran Sharma

**Affiliations:** 1 Ahmedabad, India; 2 Singapore, Singapore; 3 World Health Organisation, Geneva, Switzerland; 4 World Health Organisation, New Delhi, India

**Keywords:** Adolescent health, Peer education, RKSK, ARSH strategy, Rapid programme review

## Abstract

**Background:**

Recognizing the potential of the country’s large youth population and the importance of protecting and supporting its health and well-being, the Government of India committed to strengthening its programmes and systems for adolescents, initially through the Adolescent Reproductive and Sexual Health Strategy (ARSH) launched in 2005 and, subsequently, through the National Adolescent Health Programme (Rashtriya Kishore Swaasthya Karyakram or RKSK) launched in 2014. In 2016, in response to a request from the Government of India, the World Health Organisation undertook a rapid programme review of ARSH and RKSK at the national level and in four states (Haryana, Madhya Pradesh, Maharashtra and Uttarakhand) to identify and document lessons learnt in relation to four domains of the programmes (governance, implementation, monitoring and linkages) that could be used to enhance current and future adolescent health programming in India.

**Methodology and findings:**

A rapid programme review methodology was utilised to gain an overview of the successes and challenges of the two adolescent health programmes. A desk review of policy statements, Program Implementation Plans (PIPs) (Program Implementation Plan (PIP) is an annual process of planning, approval and allocation of budgets of various programmes under the National Health Mission (NHM). It is also used for monitoring of physical and financial progress made against the approved activities and budget.

), reports and data provided by the four State governments was conducted alongside 70 semi-structured interviews with health, education and NGO officials at national, state, district and block levels. Data showed that the ARSH Strategy put adolescent health on the agenda for the first time in India, though insufficient human and financial resources were mobilised to ensure maximum impact. Further, the Strategy’s focus on clinical service provision in a limited number of health facilities with a complementary focus on promoting community support and adolescent demand for them meant that services were not as easily accessible to adolescents in their communities, and in addition many were not even aware of them. Under RKSK, significant investment has been made in adequate management structures, as well as in community engagement and clinical service delivery at all levels of the health system. Monitoring the quality of service delivery remains a challenge in all four of the states, as does training of counsellors, nodal officers and other implementing partners. Additionally, further thought and action are required to ensure that peer educators are properly trained, supported and retained for the programme.

**Conclusions:**

India’s RKSK clearly integrated learning from the earlier ARSH Strategy. The findings of this review present an opportunity for the government and its partners to ensure that future investment in adolescent health programming continues to be framed around lessons learnt across India.

## Plain English summary

In the early 2000s, the Government of India recognized that its large youth population is exposed to significant health risks in the course of its transition to adulthood and lacks the knowledge and support to make informed choices and to carry out these choices to protect their health and wellbeing. The Adolescent Reproductive and Sexual Health Strategy (ARSH) (2005–2013) and subsequent Rashtriya Kishor Swasthya Karyakram (RKSK) or national adolescent health programme (2014-present) reflected the government’s commitment to protect and support adolescent health and well-being. In 2016, in response to a request from the Government of India the World Health Organisation conducted a rapid programme review (RPR) of the two strategies to identify and document lessons learnt in relation to four domains of the programmes (governance, implementation, monitoring and linkages) that could be used to enhance current and future adolescent health programming in India.

The WHO team conducted a detailed desk review (list provided in Appendix 1) and in-depth interviews with 70 stakeholders including representatives from government, non-government, United Nations organisations and academic institutions at the national level (17); state, district and block level officials (28); service providers (17) and representatives of non-government and professional organisations (8) in four States (Haryana, Madhya Pradesh, Maharashtra and Uttarakhand). It also reviewed community level activities conducted under the programme. Data were analysed across the domains of governance, implementation, monitoring and linkages.

The RPR showed that RKSK had succeeded in responding to the lessons learnt from the ARSH Strategy related to appointing dedicated human resources at state, district and block levels and establishing adolescent friendly health clinics up to primary health centre levels. There was increase in resource allocation for community level activities and consequent improvement in community engagement. However, there are questions about the quality and coverage of both health services and community-based activities. Further, inter-departmental linkages continued to challenge implementation due to lack of ownership over programme components of non-health departments, and monitoring was weak.

In conclusion, while some key challenges remained in all four domains, RKSK integrated learnings from the ARSH Strategy and built upon its successes in some programmatic areas. The review provided critical inputs for further planning and investment in adolescent health programming in the country.

## Introduction

According to India’s 2011 census, approximately 20 % of the total population is comprised of adolescents aged 10–19 years - a total adolescent population of over 253 million [1]. While some positive changes have been achieved for the adolescent population in the last decades in terms of improved sex ratio (884 in 1971 to 898 females/1000 males in 2011) and literacy rate (81% in 2001 to 90% in 2011) (https://india.unfpa.org/sites/default/files/pub-pdf/AProfileofAdolescentsandYouthinIndia_0.pdf), one fourth of all adolescents still belong to the disadvantaged Scheduled Castes or Scheduled Tribes and a high percentage of them live in rural areas with limited availability of and access to information and health services. Eight per cent of adolescent girls (15–19 years) have begun child bearing, 3 % have become pregnant with their first child and – despite an almost universal knowledge of modern contraceptive methods (> 90%) – less than a fifth of adolescent girls report using a modern method of contraception [2].

Recognizing the potential of the country’s large youth population and the importance of protecting and supporting adolescents’ health and well-being, the Government of India (GoI) called for a stronger health sector response to adolescent health in the early 2000s. In 2005, the Ministry of Health and Family Welfare (MoHFW) began the process of strengthening its programmes and systems for the advancement of adolescent health. This took shape initially in the Adolescent Reproductive and Sexual Health Strategy (ARSH Strategy, 2005–2013) and, subsequently, in the Rashtriya Kishor Swasthya Karyakram (RKSK or National Adolescent Health Programme, 2014 - present).

In 2016, the GoI requested the World Health Organisation (WHO) to undertake a rapid programme review (RPR) of ARSH and RKSK in four states (Haryana, Madhya Pradesh, Maharashtra and Uttarakhand), to identify and document lessons learnt in relation to four domains of the programmes (governance, implementation, monitoring and linkages) that could be used to enhance adolescent health programming in India. This paper presents the findings of the RPR, which sought to answer the following questions:

1. *What lessons were learnt from the ARSH Strategy?*
2. *What lessons have been learnt so far from RKSK?*
3. *How can these lessons be used to strengthen government programming for adolescent health?*

## Background

In 2005, the MoHFW of the GoI created the ARSH Strategy as a component of the National Rural Health Mission and the Reproductive and Child Health-II (RCH-II) programmes to ‘expand the scope and coverage of family welfare services’. Many states and union territories included ARSH interventions in their RCH-II PIPs to support effective and time-bound implementation of programme activities. The ARSH Strategy focused on selected aspects of health for all adolescents through a core package of preventive, promotive, and curative services [3]. For the most part, the ARSH Strategy employed a clinic-based approach focused on building the capacity of different cadres of health workers and reorienting and branding existing public health facilities as adolescent friendly health centres (AFHCs). The Strategy also set clear standards for quality improvement of services and developed guidelines to implement and monitor them. Whilst demand generation was a lesser focus, some states supplemented the Strategy with approaches to raise awareness of services and sensitize communities.

In 2014, in line with the new National Reproductive Maternal Newborn Child Health + Adolescent Health (RMNCH+A) Strategy’s commitment to a continuum of care approach, the MoHFW replaced the ARSH Strategy with RKSK. RKSK, which aims to ensure that ‘*all adolescents in India are able to realize their full potential by making informed and responsible decisions related to their health and well-being, and by accessing the services and support they need to do so*,’ [4] broadened the focus beyond sexual and reproductive health (SRH) to include non-communicable diseases, nutrition, mental health, substance misuse and injuries and violence. It employs clinic- and community-based service provision models and demand generation activities. Implementation of RKSK is currently underway, with a special focus on 213 districts across the country.

## Methodology

Rapid programme reviews (RPR) provide guidance on what implementing agencies can do to build on their strengths and tackle areas of weakness in a manner that is typically less time and cost intensive than regular programme reviews [5]. Given time and financial resource constraints, RPR was chosen as the methodology for this review. The review was conducted in two phases: Phase I was conducted at the national level, and Phase II was conducted in two districts each in four states (Haryana, Madhya Pradesh, Maharashtra and Uttarakhand) selected in consultation with the MoHFW and the WHO India Office. This paper primarily presents the composite findings of these four states.

### Data collection

Data for the RPR was collected through a desk review of third party studies and policy documents, guidelines, PIPs, reports and data from the MoHFW. A list of documents reviewed is provided in Appendix 1. Haryana, Maharashtra and Uttarakhand provided data on the number of AFHCs made functional; adolescent counsellors employed; medical officers, auxiliary nurse midwives (ANMs) and peer educators (PEs) trained; adolescent health days (AHDs) held; quarterly service use, Weekly Iron Folic acid Supplementation (WIFS) coverage, and annual budget and expenditure. However, data was not available for all of these indicators for all districts and time periods. Madhya Pradesh was not able to provide these data as RKSK officials at both state and district levels did not have up-to-date consolidated data on the relevant indicators available for sharing. Reports from IPE Global^1^ were also consulted for Uttarakhand and Haryana, as were evaluations, research studies and academic papers for all four states.

The findings from the desk review were complemented with semi-structured interviews to explore the views, opinions and experiences of a range of stakeholder related to four domains of the programmes: governance, implementation, monitoring and linkages. Interviews were conducted with 70 stakeholders including representatives from government, non-government, United Nations organisations and academic institutions at the national level (17); state, district and block level officials (28); service providers (17) and representatives of non-government and professional organisations (8) in four states (list provided in Appendix 2). Additionally, informal discussions were carried out in each state with frontline workers, such as ANMs, accredited social health activists (ASHAs), counsellors and PEs during visits to AFHCs, AHDs and adolescent health clubs (AHCs). Informed consent was obtained from all individual participants included in the study. All the interviews were conducted face to face by a trained researcher/social scientist with more than 25 years experience of qualitative research. A semi-structured interview field guide was used for conducting the interviews. Interviews notes were transcribed and translated (in case of service providers, frontline workers and PEs, as these were conducted in the local language) by the researcher. Interviews with all senior officials were conducted in English.

### Data analysis

The data were analysed across the following four domains:

- *Governance*: findings related to centralized planning for the ARSH Strategy and RKSK management, human resources, budget and procurement processes;
- *Implementation*: findings related to the roll-out of ARSH and RKSK interventions and approaches. For RKSK, this domain was further subdivided in the key programmatic components - AFHCs, counselling, AHDs, peer education, WIFS and Menstrual Hygiene Scheme (MHS);
- *Monitoring and evaluation*: findings related to the use of data for programme planning or quality improvement;
- *Linkages*: findings related to the involvement of non-governmental organizations (NGOs) and other government departments.

These four domains are typical of those examined through a RPR, though they were adapted through an iterative process involving all of the authors following the initial desk review, as well as an assessment of the available data. Key indicators and accompanying research questions were identified following the initial desk review, and these formed the basis of the interview guides that were used with the key informants. Thematic analysis was used to identify patterns of meaning in the key informant interview transcripts. Additionally, successes and challenges for each of these four dimensions and their key components were examined.

## Findings

### Governance

The ARSH Strategy put adolescent health on the agenda for the first time in India; this was in part due to the establishment of dedicated AFHCs and community engagement through NGO partners. However, programme planning appears to have been weak, and human and financial resources were not deployed in an optimal manner. For example, NGOs were contracted in some states, but their engagement was discontinued mid-way through the programme with a change in government. To a large extent, the ARSH Strategy remained in ‘project-mode’ throughout its life span, an assessment that reflects the challenges in integrating it fully into the broader health system. From the available data, adolescents do not appear to have been involved in its governance.

RKSK’s design incorporated learning from the ARSH Strategy, particularly in relation to the need for adequate human and financial resources. A clear organogram was developed to define the human resources required for each programmatic component from state to block level. Despite this new standardized framework, key positions remained vacant at state and district levels at the time of the RPR, and most nodal officers at both these levels had a number of other responsibilities that they considered as important as RKSK. Adequate budgets were allocated at the state level for implementation of RKSK, but rules and regulations on their use were considered by key informants to be too rigid, thus limiting the responsiveness of the programme to the different needs across times and places. Likewise, programme activities were not well coordinated; for example, in several cases, training on the Menstrual Health Scheme (MHS) occurred months before necessary commodities, such as sanitary pads, had been secured. As with the ARSH Strategy, adolescents do not appear to have been involved RKSK’s governance.

### Implementation

The ARSH Strategy focused solely on the provision of SRH services to adolescents through AFHCs operating at block or higher levels of the health system. ANMs, ASHAs (wherever available) and PEs were employed to drive demand amongst adolescents, though incentive and support structures for these cadres were lacking. In some states, NGOs were actively involved in community engagement activities. However, there were still challenges that affected the ability of adolescents to access services, including cultural resistance, distance to AFHCs and absence of follow-up services at the community level.

RKSK was designed to be more comprehensive, with SRH being addressed within a broader approach to adolescent health. This, alongside the change in name, helped pave the way for greater acceptability amongst health providers, and amongst communities and adolescents, themselves. The scale-up of community engagement and demand generation activities with expanded access to AFHCs and counselling services, has led to increased service use in some districts. Uttarakhand, Haryana and Maharashtra saw increases in service use during the financial year 2016–2017 as compared to the previous year, whilst Madhya Pradesh saw a sizeable decrease.

#### Adolescent friendly health clinics

Through RKSK, AFHCs have been integrated into district and sub-district hospitals, community health centres (CHCs) and some primary health centres (PHCs). At the clinics, adolescents are able to access counselling services, health services, and referrals for other specialist needs. Whilst RKSK has attempted to bring services ‘closer to home’ for adolescents than they were under the ARSH Strategy, many still have difficulties in accessing services due to geographical barriers. Further, capacity-building for ASHAs and ANMs under RKSK has been prioritized over that of facility medical officers, whose buy-in is crucial to ensuring the RKSK’s continued success. As with governance, young people are not afforded a role within the AFHCs.

This RPR found evidence that the Protection of Children from Sexual Offenses Act is creating confusion amongst providers regarding the provision of SRH services to adolescents. The Act criminalizes sexual acts with minors (people under age 18) and makes no exception for consensual sexual relationships between minors. It mandates that those with knowledge of such offenses report them to the relevant authorities, under threat of imprisonment. Given these reporting restrictions, it appears that providers are inclined to deny SRH services to young people in some states.

#### Counselling

Counselling services in clinics, schools, and community settings have been strengthened and expanded under RKSK. While many female and male counsellors dedicated to adolescent health were recruited and trained in the first 3 years of RKSK, vacancies remained in almost every district; this was particularly true for male counsellors. Apart from the initial trainings for adolescent health counsellors, no refresher trainings were carried out. Further, without an adequate monitoring system in place, it was not possible to measure the quality and content of the counselling provided. However, there is evidence that counsellors’ heavy caseloads at the AFHCs and the lack of transport allowance and corresponding security arrangements negatively impacted their outreach services, particularly to hard-to-reach areas.

#### Adolescent health days

AHDs is a novel mode of connecting adolescents with services and of engaging parents, community leaders and young people, through RKSK. The AHDs are organized and facilitated by ANMs, ASHAs, PEs, and counsellors. However, there is evidence that the lack of a structured plan for the AHDs meant that they were implemented in an ad hoc manner across the four states. Similarly, outcomes for AHDs are not clearly defined nor monitored.

#### Peer education

Through RKSK, PEs are meant to engage with young people at the community level, including at AHDs and monthly AHCs. Thousands of PEs were recruited and completed initial trainings in the four states. In one state, PEs have been using innovative technology-based information, education and communication (IEC) materials for community education. In most states, PEs are being supervised and supported on a monthly basis at the AHCs by ANMs who are based at sub-centres. These ANMs are oriented on how to mentor and support the PEs. Adolescent health counsellors are also involved in responding to the PEs’ questions. Across several states, there were doubts about whether the methods used to recruit PEs were appropriate, and there were concerns about the adequacy of their training and support. Likewise, a lack of monitoring systems to track PEs’ activities leaves questions about their reach in the communities where they operated. There is evidence that the absence of a transport allowance or financial incentive is affecting PEs’ motivation and retention rates, and that cultural and parental objections negatively impact their involvement.

#### Weekly iron folic acid supplementation

The MoHFW launched the WIFS programme in 2012 to respond to the high prevalence of anaemia amongst adolescents through supervised weekly ingestion of an Iron Folic Acid (IFA) supplement and biannual helminthic control. When RKSK was launched, WIFS was included as one of the key components of the programme. The WIFS component relies heavily on partnership with the Education and Women and Child Development Departments. In most states, government school teachers within programme catchment areas have been oriented on the provision of the tablets to students. However, reluctance amongst teachers to dispense the tablets remains one of the greatest challenges; this is influenced by negative publicity following the hospitalization of young people in more than one setting who experienced side effects after taking the tablets at school.

#### Menstrual hygiene scheme (MHS)

The MoHFW launched the MHS in 2011 with the aim of increasing awareness of menstrual hygiene, promoting access to and use of high quality sanitary napkins and ensuring safe disposal of sanitary napkins in an environmentally friendly manner. The MHS in its entirety was not operational in any of the four states due to procurement challenges. However, Anganwadi workers, ASHAs and ANMs provided menstrual hygiene education to adolescents through Education Department-sponsored life skills education and/or health education sessions.

### Monitoring

Under ARSH, a structured monitoring system was established for clinic-based services and a number of third party evaluations were conducted. Monitoring was largely administrative and it seems unlikely from the evidence available for this review that the limited data collected were used for programme planning or quality improvement. Documentation was limited in all states, resulting in a dearth of evidence and data for this review.

Under RKSK, a structured monitoring system for clinic- and community-based services was established, from community to state and national levels. Several states are using community feedback, third party surveys and innovative mechanisms, such as video conferencing and WhatsApp, for monitoring and troubleshooting, indicating intentional usage of data for programme planning and quality improvement. Nonetheless, most monitoring efforts focus on administrative indicators, such as the number of trainings conducted and clients served. The programme lacks insight on the quality of the services and activities provided, including from adolescents’ perspectives. There are specific challenges with WIFS related to the separation of health and education monitoring systems; these challenges manifest in the tardy submission of incomplete reports. No data were available for some components of RKSK, including mental health, substance misuse and injuries and violence.

### Linkages

There was no evidence of linkages with other state departments during the implementation of the ARSH Strategy. Partnerships existed with local NGOs that had on-going programmes for adolescents; however, challenges with these partnerships led to their discontinuation in most states.

‘Convergence’ is a key strategy within RKSK, as set out in the programme’s operational framework. In all states, attempts have been made to forge partnerships with various government departments, including Education, Women and Child Development and Panchayati Raj.^2^ In Madhya Pradesh, NGOs were systematically involved in community engagement and service delivery activities, whilst their involvement in the other three states was ad hoc. Challenges with interdepartmental collaboration were identified in all four states. In particular, it was evident that the Education Departments were not completely on board; as a result, its concerns – particularly with WIFS – went unaddressed and teachers remained wary of being held accountable for negative health outcomes. Efforts to institutionalize convergence have stalled due to the lack of formalized structures for other departments’ engagement, apart from attendance at quarterly RKSK meetings.

## Discussion and recommendations

This review set out to identify lessons learnt from the ARSH Strategy and RKSK to assist the MoHFW in identifying areas and strategies to strengthen RKSK going forward. Data were collected, analysed and presented according to four domains: governance, implementation, monitoring and linkages. The findings indicate that RKSK improved upon the ARSH Strategy’s governance model by creating a human resources framework that ensured nodal officers exist at state, district and block levels and responded to the recommendations related to geographic barriers to access by establishing AFHCs right up to CHC and PHC levels. There has been notable improvement in community engagement, with resources being allocated to AHDs, peer education and integration with the work of existing frontline workers. However, monitoring and linkages remain challenges. In part, this is due to different government departments being responsible for overseeing the execution of different components and the consequential lack of ownership that they feel over components they are not directly responsible for and for adolescent health programming as a whole.

Based on these findings, WHO made domain-wise recommendations, which are presented in Table 1 below:

**Table 1:** Domain-wise recommendations emerging from the rapid programme review

Domain	Recommendation
**Governance**	• Develop a recruitment and retention strategy to ensure that all crucial vacancies are filled. • If pre-existing personnel are to be asked to take on RKSK-related responsibilities, ensure that they have both the space in their agendas and the competencies to do so. • Allow flexibility between budget lines, allowing implementers to reallocate funding on a more responsive basis. • Ensure procurement processes allow for the timely and adequate supply of all commodities, including sanitary pads. • Align activities so that they are synergistic (i.e. trainings and disbursement).
**Implementation**	**a. AFHCs:** • Introduce training for medical officers on adolescent health and their role in the implementation of RKSK to ensure their buy-in and ownership of the programme. • Ensure follow up with training, retraining, provision of desk reference tools, supportive supervision, and collaborative learning. • Conduct an analysis of the reach of clinical and counselling services to deepen understanding of which adolescents are / are not receiving them **b. Counselling:** • Ensure follow up training, supportive supervision, and collaborative learning. • Ensure adequate budget is allocated to compensate and to ensure the safety of counsellors for work-related travel. **c. AHDs:** • Provide further guidance to RKSK districts on the structure of AHDs. • Define the intended outcomes and put in place a monitoring system to measure them. **d. Peer education:** • Conduct further research on effective IEC materials and consider scaling up best practices. • Provide more support to PEs with training and supportive supervision, and design activities to increase support in their communities from parents and leaders. • Work with PEs to develop more effective recruitment and incentive schemes. • Ensure adequate budget allocation to compensate and to ensure the safety of PEs for work-related travel. **e. WIFS:** • Work with teachers and principals to determine what support they need and develop a responsive strategy. • Involve community groups to generate support for WIFS. **f. MHS:** • Revisit the procurement procedures to find out blockages and ensure corrective action.
**Monitoring**	• Extend monitoring beyond administrative indicators to outputs and outcomes. • Build on fledgling efforts to institutionalize mechanisms to utilize data in programme monitoring, evaluation and improvement efforts. • Train nodal officers at all levels on the use of data for quality and programme improvement
**Linkages**	• Develop a formal strategy to build meaningful convergence with other governmental departments and NGOs with clear mechanisms, roles, and responsibilities. Monitor this convergence on an on-going basis, with corrective action.
**Cross-cutting**	• Ensure the participation and inclusion of young people in all elements of RKSK, including governance, implementation and monitoring.

These recommendations emerged after data collection and analysis and were based on a comprehensive overview of the programme. The recommendations were developed through an iterative process of discussion amongst the authors as well as other colleagues involved in the RKSK programme who – at one time or another – provided input into the study and/or manuscript. These are presented as composite recommendations, as any attempt to filter these according to the exact source of the relevant data would have been artificial.

Findings from other assessments of RKSK echo many of the key findings of this review. An impact assessment of the project ‘Strengthening RKSK through Government Civil Society Partnership in West Bengal’ reported that community sensitization efforts for parents and other stakeholders created a favourable environment for adolescents [6]. A review of RKSK in Sitamarhi district, Bihar showed that whilst community engagement and operationalization of AFHCs at CHCs are notable strengths, the absence of dedicated counsellors and the lack of incentives for PEs were challenges for the programme [7]. Further, a research project entitled ‘Understanding the lives of adolescents and young adults’ in Uttar Pradesh highlighted programme challenges such as lack of timely procurement of supplies, difficulties with recruiting counsellors and PEs, insufficient training and support for frontline workers on how to respond to the needs of adolescents, and lack of disaggregated data to assess the quality and reach of services [8]. Finally, a study in two districts in Gujarat found that RKSK implementing officers required greater clarity on the content of the programme, and that RKSK was not a top priority for district officials [9].

There were findings from other studies, however, that departed from the findings of this review. In the West Bengal study, the involvement of NGOs was found to facilitate innovation and convergence across different stakeholders, whereas this review found that NGOs were not involved in any systematic way in three of the four states [6]. A study in Haryana showed that the implementation of WIFS and MHS components were successful, whereas this review found that these components experienced challenges due to lack of support and procurement processes [9]. Finally, the Gujarat study found that financial resources for RKSK were not sufficient, a finding at odds with this review [10].

Based on the findings and recommendations of this review, the MOHFW engaged WHO’s support to develop a concerted strategy to strengthen RKSK’s implementation, and to draw out lessons from this process. In response, WHO and the MOHFW, with UNICEF and UNFPA, designed a Learning District initiative, whereby mentoring NGOs and academic institutions provide technical support to State and District Programme Management Units in ten districts, to design, implement and monitor a context-specific (needs- and capacity-based) package of health and social interventions in line with the operational guidelines of RKSK. The initiative includes provisions for strong on-going monitoring and nested implementation research to assess the feasibility, acceptability and effectiveness of implementing RKSK at scale, with quality and equity, and to understand how challenges and bottlenecks can be addressed in a practical and systematic manner. The Learning District initiative was launched by the MOHFW in July 2018.

## Conclusions

Whilst RKSK clearly integrated learning from the ARSH Strategy and built upon its successes in some programmatic areas, important challenges remain in all four domains: governance, implementation, monitoring and linkages. The findings of this review and the subsequent launch of the Learning Districts initiative present an opportunity for the MoHFW and its partners at all administrative levels to ensure that future planning for and investment in adolescent health programming across India is framed around the successes and challenges of the past 12 years and contributes to progress for years to come.

### Limitations

The range of key informants included in the review provides a broad overview of successes and challenges, although the sample size is a limitation. Few key informants were able to comment meaningfully on the ARSH Strategy given the high turnover in staff members; documentation of the Strategy was also missing due to lack of structures for institutional memory. Another limitation of this review was the lack of young people’s perspectives. Likewise, access to officials from other state-level departments, including the Education Departments, was limited due to their unavailability. Whilst every effort was made to gather and review all the relevant documents, it is possible that key documents that contained useful information were inadvertently omitted.

## Data Availability

While material and data in public domain will be available if requested, raw data or transcripts of key informant interviews will not be available as per the informed consent agreements with them.

## Appendix 1

**List of Documents Reviewed.**

**Table:**

Sr. No.	Documents
1	ARSH Implementation Guidelines for State and District Programme Managers. Available at: https://nhm.gov.in/New_Updates_2018/NHM_Components/RMNCHA/AH/guidelines/Implementation_Guidelines_Rashtriya_Kishor_Swasthya_Karyakram(RKSK)_2018.pdf
2	Adolescent Health Division, Ministry of Health and Family Welfare. New Delhi. 2014. *Guidelines for implementation of RKSK.*
3	Investing in Young People Evaluative Evidence from 10 UNFPA Country Programmes. Available at: https://www.unfpa.org/sites/default/files/pub-pdf/Investing%20in%20YP_Evaluative%20Evidence%20from%2010%20CPEs_OSQA%20Branch%20Programme%20Division_July%202012.pdf
4	Adolescence An Age of Opportunity. Available at: https://www.unicef.org/publications/files/SOWC_2011_Main_Report_EN_02242011.pdf
5	Adolescent and youth reproductive health in India: Status, issues, policies and programmes. Available at: https://hivhealthclearinghouse.unesco.org/library/documents/adolescent-and-youth-reproductive-health-india-status-issues-policies-and-programs
6	Adolescents in India: A desk review of existing evidence and behaviours, programmes and policies. Available at: http://4dj7dt2ychlw3310xlowzop2.wpengine.netdna-cdn.com/wp-content/uploads/2016/09/Adolescents_in_India.pdf
7	Studies on adolescent girls: Analytical review. Available at: https://www.nipccd.nic.in/file/reports/eag.pdf
8	1st to 8th Joint Review Mission. Aide Memoire. Available at: https://nhm.gov.in/index1.php?lang=1&level=1&sublinkid=1237&lid=201.
9	7th to 9th Common Review Mission reports. Available at: http://nhsrcindia.org/category-detail/common-review-mission-(crm)-reports/MTE1
10	International Institute for Population Sciences (2005–2006) A Profile of Youth in India. Available at: https://dhsprogram.com/pubs/pdf/OD59/OD59.pdf
11	Ministry of Health and Family Welfare: Five Years (2009–14) Achievements and New Initiatives. Not available online.
12	Centre for Advanced Research & Development (2009–2010) Impact Assessment of ICDS in Madhya Pradesh. Available at: http://mpplanningcommission.gov.in/ international-aided projects/pmpsu/publication/Final%20Study %20Report%20%20 on%20Impact%20Assessement%20%20of%20%20ICDS%20in%20M.P%20(English).pdf
13	Population Council (2010) Youth in India: Situation and Needs 2006–2007. Available at: http://www.popcouncil.org/uploads/pdfs/2010PGY_YouthInIndiaExecSumm.pdf.
14	Centre for Innovations in Public Health (2011) UDAAN: Uttarakhand. Available from: http://www.cips.org.in/documents/DownloadPDF/downloadpdf.php?id=111&category=Health
15	USAID (2012) Promoting Adolescent Reproductive Health in Uttarakhand and Uttar Pradesh, India. Available at: http://pdf.usaid.gov/pdf_docs/pnadz546.pdf; Government of Uttarakhand (no date) Rashtriya Kishor Swasthya Karyakram. Available at: http://ukhfws.org/details.php?pgID=mn_2568
16	Ministry of Health and Family Welfare (2012–2013) District Level Household and Facility Survey 4: State Fact Sheets for Haryana, Maharashtra, Madhya Pradesh and Uttarakhand. Available at: heetp://rchiips.org/pdf/dlhs4/repor. Last access 9 October 2017
17	Haryana Health Department (2012–2013) *Guidelines for ARSH Programme for the year 2012–13.* Not available online.
18	Ministry of Health and Family Welfare Annual Reports (2013–14). Available at: https://mohfw.gov.in/annual-report-department-health-and-family-welfare%2D%2D2013-14
19	Ministry of Health and Family Welfare, Government of India (2014) *RKSK Operational Framework: Translating strategy into programmes*. Available at: <http://www.expandnet.net/PDFs/India_RKSK_Strategy%20Operational_Framework_2014.pdf> Last accessed 21 September 2017.
20	Ministry of Health and Family Welfare Annual Reports (2015–16). Available at: https://mohfw.gov.in/annual-report-department-health-and-family-welfare%2D%2D2015-16
21	Ministry of Health and Family Welfare (2015–16) National Family Health Survey - 4, Fact Sheets: Haryana, Maharashtra, Madhya Pradesh and Uttarakhand. Available at: http://rchiips.org/NFHS/pdf/NFHS4/
22	Ministry of Health and Family Welfare. 2016. WIFS Report 2015–2016. Not available online.
23	Ministry of Health and Family Welfare. 2017. WIFS Report 2016–2017. Not available online.
24	Ministry of Health and Family Welfare. 2017. *Funds approved for decentralized procurement of Sanitary Napkins under Menstrual Hygiene Scheme* (FY 2016–17). Not available online.
25	Ministry of Health and Family Welfare. 2017. Menstrual Hygiene Report. Not available online.
26	Haryana State Government (2017) RKSK PIP 2017–2018. Not available online.
27	Joshi, B.N., Chauhan, S.L., Kulkarni, R.N., Kamlapurkar, B. and Mehta, R. (2017) Operationalizing Adolescent Health Services at Primary Health Care Level in India: Processes, Challenges and Outputs. Health, 9, 1–13. 10.4236/health.2017.91001
28	State Programme Implementation Plans. Available at: http://pip.nhm.gov.in/

## Appendix 2

**List of key informants.**

**Table:**

National Level	
1–4	Ministry of Health and Family Welfare: 2 officials and 2 consultants
5	National Institute for Research in Reproductive Health
6	CHETNA
7	CINI
8	MAMTA
9	Centre for Catalyzing Change
10	Engender Health
11	International Centre for Research on Women
12	Pathfinder (previously employed.)
13	Lady Hardinge Medical College/Kalawati Saran Children’s Hospital
14–15	WHO, SEARO and CO: 2 representatives
16–17	UN Organizations: 2 representatives: UNFPA, UNICEF
**Haryana**	
18	State Level RKSK official
19	State Level RKSK consultant
20	Programme Official, WIFS, MHS, NDD
21	IPE Global Coordinator (RMNCHA)
22	State Level WCD Official, WCD Programme Officer
23	Deputy Chief Medical Officer, District 1
24	District Adolescent Programme Officer, District 1
25	Adolescent health counsellor, District 1
26	ARSH Nodal Officer
27	FOGSI Representative
28	FPAI Representative
29	SWACH Representative
30	District Adolescent Programme Officer, District 2
31	Adolescent health counsellor, District 2
32	Deputy Chief Medical Officer, District 2
33	Block Development Officer, District 2
**Madhya Pradesh**	
34	Chief Medical Officer, District 1
35	Chief Medical Officer, District 2
36	Adolescent health counsellor, District 1
37	Adolescent health counsellor, District 2
38	Civil surgeon, District 1
39	Civil surgeon, District 2
40	District Community Mobilizer, District 1
41	State Level Adolescent Health Official
42	Representative, Samarthan NGO
43	District Community Mobilizer, District 2
**Maharashtra**	
44	State Level Official, Family Health and Welfare
45	State Level Official, RKSK
46–47	Group Interview: District Reproductive and Child Health Officer (RCHO), District Health Officer (DHO), Civil Surgeon (CS) and Consultant Paediatrician
48–49	Group Interview: District Reproductive and Child Health Officer (RCHO), District Health Officer (DHO), Civil Surgeon (CS)
50	Managing Trustee, Institute of Health Management
51	Academic, National level research institution
52	State RKSK Consultant
**Uttarakhand**	
53	State Level RKSK Consultant
54	State Level RKSK Official
55–56	Additional Chief Medical Officer, District 1 and 2
57–58	MO-in-charge,, District 1and 2
59–60	2 Adolescent health counsellors, District 1
61	FOGSI Representative
62	State Level Official, health services
63	RKSK Programme Officer
64	District Level Adolescent Programme Official
65	State Level Official, Department of Education
66	NGO representative
67–70	3 Adolescent health counsellors, District 2

## Abbreviations

ADH: Adolescent Health Day
AFHC: Adolescent Friendly Health Clinics
AHC: Adolescent Health Club
ANM: Auxiliary Nurse Midwife
ARSH: Adolescent Reproductive and Sexual Health Strategy
ASHA: Accredited Social Health Activist
CHC: Community Health Centre
GoI: Government of India
IEC: Information, Education and Communication
IFA: Iron Folic Acid
MHS: Menstrual Hygiene Scheme
MoHFW: Ministry of Health and Family Welfare
NGO: Non-Government Organisation
PE: Peer Educator
PHC: Primary Health Centre
PIP: Project Implementation Plan
RCH-II: Reproductive and Child Health-II
RKSK: Rashtriya Kishore Swaasthya Karyakram
RMNCH+A: Reproductive Maternal Newborn Child Health + Adolescent Health
RPR: Rapid Programme Review
SRH: Sexual and Reproductive Health
SRHR: Sexual and Reproductive Health Rights
WHO: World Health Organisation
WIFS: Weekly Iron Folic acid Supplementation

## Footnotes

1 IPE Global Limited is an Indian international development consulting group that is providing technical assistance to the state government of Haryana.

2 Panchayati Raj refers to the system of local self-government in India
